# Supplementary material for: MetaRibo-Seq measures translation in microbiomes
Source: Nat Commun. 2020 Jun 29;11:3268. doi: 10.1038/s41467-020-17081-z (PMC7324362; doi:10.1038/s41467-020-17081-z)
Supplement: Supplementary file 10 — Supplementary Data 7 [file 41467_2020_17081_MOESM10_ESM.zip › File2/Confidence_High_Taxonomy/377283_out.krona.html]

Javascript must be enabled to view this page.

members
magnitude
magnitudeUnassigned
count
unassigned
taxon
rank

377283\_out

68

2
68
superkingdom

1239
68
phylum


SRS011084\_contig\_number\_30633SRS011302\_contig\_number\_18031SRS013098\_contig\_number\_9555SRS013521\_contig\_number\_2969SRS014235\_contig\_number\_contig-100\_3065.253403SRS015960\_contig\_number\_14423SRS017821\_contig\_number\_contig-100\_3425.36868SRS018817\_contig\_number\_14524SRS018836\_contig\_number\_11555SRS019445\_contig\_number\_23217SRS020233\_contig\_number\_44509SRS020394\_contig\_number\_9558SRS020869\_contig\_number\_28046SRS021948\_contig\_number\_31026SRS024435\_contig\_number\_30807SRS046369\_contig\_number\_9950SRS047433\_contig\_number\_9071SRS048870\_contig\_number\_15489SRS049446\_contig\_number\_9602SRS049959\_contig\_number\_16261SRS049995\_contig\_number\_contig-100\_5162.173221SRS052697\_contig\_number\_45747SRS053356\_contig\_number\_11066SRS054059\_contig\_number\_4709SRS054905\_contig\_number\_1432SRS055017\_contig\_number\_37097SRS056259\_contig\_number\_5820SRS063985\_contig\_number\_24661SRS064276\_contig\_number\_31117SRS065397\_contig\_number\_contig-100\_3430.78040SRS075984\_contig\_number\_21725SRS077024\_contig\_number\_6211SRS077294\_contig\_number\_14564SRS078176\_contig\_number\_18231SRS078242\_contig\_number\_1732SRS1041031\_contig\_number\_20040SRS1041032\_contig\_number\_11046SRS1041039\_contig\_number\_20121SRS1041137\_contig\_number\_14523SRS1041140\_contig\_number\_20839SRS1041145\_contig\_number\_21004SRS1041157\_contig\_number\_contig-100\_2347.48352SRS104400\_contig\_number\_38263SRS104975\_contig\_number\_contig-100\_3154.50370SRS142503\_contig\_number\_17932SRS142542\_contig\_number\_578SRS143417\_contig\_number\_24141SRS143895\_contig\_number\_1721SRS144135\_contig\_number\_5833SRS144506\_contig\_number\_42476SRS144537\_contig\_number\_contig-100\_3139.241527SRS146812\_contig\_number\_22931SRS146813\_contig\_number\_29683SRS147346\_contig\_number\_29513SRS148196\_contig\_number\_19741SRS148817\_contig\_number\_1931SRS149784\_contig\_number\_7407SRS150029\_contig\_number\_13976SRS892882\_contig\_number\_13362SRS893366\_contig\_number\_10934SRS893373\_contig\_number\_14420SRS893383\_contig\_number\_18919SRS971276\_contig\_number\_25759
1263007
63
species

186801
5
class

5
186802
order

species
1950886

SRS148159\_contig\_number\_30455
1

family
4
216572

genus
4
459786

1945593

SRS015663\_contig\_number\_22899SRS077392\_contig\_number\_964SRS144506\_contig\_number\_13467
3
species

1
1897011

SRS075773\_contig\_number\_13582
species
